# Outbreak of *Corynebacterium diphtheriae* among asylum seekers in Belgium in 2022: operational challenges and lessons learnt

**DOI:** 10.2807/1560-7917.ES.2023.28.44.2300130

**Published:** 2023-11-02

**Authors:** Stéphanie Jacquinet, Helena Martini, Jean-Paul Mangion, Sarah Neusy, Aurélie Detollenaere, Naïma Hammami, Lien Bruggeman, Bart Hoorelbeke, Denis Pierard, Laura Cornelissen

**Affiliations:** 1 Epidemiology of infectious diseases, Department of Epidemiology and Public Health, Sciensano, Brussels, Belgium; 2 Department of Microbiology, National Reference Centre for toxigenic corynebacteria, Universitair Ziekenhuis Brussel, Vrije Universiteit Brussel (VUB), Brussels, Belgium; 3 Médecins Sans Frontières, Brussels, Belgium; 4 Preventive Medecine, Commission Communautaire Commune, Brussels, Belgium; 5 Agency for Care and Health, Team Infection control and prevention, Brussels, Belgium; 6 National Medical Coordinator, Fedasil, Brussels, Belgium; 7 Public Health Emergencies department, Federal Public Service – Health, Food Chain Safety and Environment, Brussels, Belgium

**Keywords:** *Corynebacterium* *diphtheriae*, outbreak management, asylum seekers, cutaneous diphtheria, European countries

## Abstract

Since 2022, European countries have been facing an outbreak of mainly cutaneous diphtheria caused by toxigenic *Corynebacterium diphtheriae* among asylum seekers. In Belgium, between 1 March and 31 December 2022, 25 cases of toxigenic *C. diphtheriae* infection were confirmed among asylum seekers, mostly among young males from Afghanistan. Multi-locus sequence typing showed that most isolates belonged to sequence types 574 or 377, similar to the majority of cases in other European countries. The investigation and management of the outbreak, with many asylum seekers without shelter, required adjustments to case finding, contact tracing and treatment procedures. A test-and-treat centre was organised by non-governmental organisations, the duration of the antimicrobial treatment was shortened to increase compliance, and isolation and contact tracing of cases was not possible. A vaccination centre was opened, and mobile vaccination campaigns were organised to vaccinate a maximum of asylum seekers. No more cases were detected between end December 2022 and May 2023. Unfortunately, though, three cases of respiratory diphtheria, including one death, were reported at the end of June 2023. To prevent future outbreaks, specific attention and sufficient resources should be allocated to this vulnerable population, in Belgium and at international level.

**Key public health message**

**What did you want to address in this study?**

Diphtheria is a bacterial disease caused by certain strains of *Corynebacterium diphtheriae* that produce diphtheria toxin. The disease is transmitted between humans. In 2022, an unusual outbreak of diphtheria occurred among asylum seekers in Europe, including Belgium. We describe the outbreak investigation and the measures taken to control the outbreak so that we can learn lessons for the future.

**What have we learnt from this study?**

The lack of shelter for many asylum seekers made outbreak management very complex. Our best option to prevent severe cases was thus to vaccinate as many asylum seekers as possible, reaching out to people living on the streets through a mobile vaccination team. Co-operation with non-governmental organisations was crucial to offer treatment to possible cases of diphtheria with skin lesions in asylum seekers without shelter.

**What are the implications of your findings for public health?**

To prevent future outbreaks, specific attention and sufficient resources should be allocated to asylum seekers in Belgium and in Europe, including rapid vaccination at arrival. Managing an outbreak in a population living in precarious conditions, such as asylum seekers, is challenging and requires a pragmatic adjustment of existing guidelines and procedures.

## Background

Diphtheria is a disease that can present as a skin infection (cutaneous diphtheria), as a mild upper respiratory tract infection, or as severe pseudomembranous respiratory diphtheria [1,2]. Humans are the only reservoir, and the principal modes of transmission are airborne respiratory droplets or direct contact with cutaneous lesions or fomites [1,2]. It is caused by toxin-producing strains of *Corynebacterium diphtheriae,* and the incubation period ranges from 2 to 5 days but can exceed 10 days.

The morbidity and mortality of diphtheria are caused by the diphtheria toxin, encoded by the *tox* gene, which is responsible, among other factors, for systemic complications such as myocarditis and neuritis [1]. The diphtheria toxin can also be produced by the zoonotic species *C. ulcerans* and *C. pseudotuberculosis*, but these are rarely the cause of severe disease in humans [2].

The only treatment available to counter the effects of the toxin is equine diphtheria antitoxin (DAT), which should be administrated within 48 hours of the initial symptoms. However, DAT production, supply and availability have declined the last decade because of very low demand in Europe and many European countries have experienced shortages [3,4]. Full vaccination (≥ 3 doses) is 87% effective against symptomatic disease and 93% effective in preventing death [5]. The toxoid vaccine provides protection against local and systemic effects of the toxin, but vaccinated individuals can still be colonised, become asymptomatic carriers and transmit the bacterium [5]. Antimicrobial treatment is needed to eliminate the bacterium, halt toxin production and reduce transmissibility [1].

In the European Union (EU) countries, the majority of cases of *C. diphtheriae* infection reported in the last years have been cutaneous and imported from African, Eastern Mediterranean and South-East Asian regions [3,6]. Between 2016 and 2020, 128 cases of *C. diphtheriae* infection were reported in EU countries and 29 cases in 2021 [3,7]. In Belgium, cases of *C. diphtheriae* infection are very rare and often travel-related. Between 2016 and 2021, only four cases were notified [7,8]. The last reported death related to a *C. diphtheriae* infection in Belgium occurred in 2016 in an unvaccinated 3-year-old girl presenting cardiac complications caused by the toxin [9]. In 2022, vaccination coverage among infants in the EU was 94% for the third dose of diphtheria, tetanus toxoid and pertussis vaccine (DTP3) [10]. In Belgium, in 2022, coverage of DTP3 was 98% [10].

In high-income countries, diphtheria has become a rare and forgotten disease. However, small outbreaks of cutaneous diphtheria have been previously described among asylum seekers arriving in Europe [11,12]. This population is particularly at risk of infectious diseases because of their generally low vaccination coverage [13], poor hygienic conditions on their travel routes and crowded living conditions in refugee camps. Around 330,000 irregular border crossings were detected at EU’s external border in 2022, with 45% of all irregular entries occurring via the Western Balkans [14]. Around 966,000 asylum applications were filed in European countries in 2022, the highest number since 2016 [15]. Syrians and Afghans represented a large group of asylum seekers, with 132,000 and 129,000 asylum applications lodged, respectively. Hosting and caring for asylum seekers are challenging for the authorities.

Since 2022, European countries such as Austria, France, Germany, Norway, Switzerland, the United Kingdom (UK) and Belgium, have been facing an outbreak of cutaneous diphtheria caused by *C. diphtheriae* among asylum seekers, mainly among young males from Afghanistan and Syria [3,16,17]. Cases of respiratory diphtheria have also been reported, including fatal cases.

In 2022 in Belgium, 36,871 people applied for international protection, an increase by 42% compared with 2021. Afghans and Syrians represented the two most important nationalities with 6,156 and 3,545 applications, respectively. Most (70.6%) applicants were males, and for some countries, such as Afghanistan, the percentage of male applicants was even higher (93.2%) [18]. The increase in the number of asylum seekers coupled with the arrival of Ukrainian refugees has overloaded the Belgian reception network. In 2022, the Federal Agency for the reception of asylum seekers (Fedasil) was convicted more than 5,000 times by Belgian courts in 2022 for failing to provide reception [19]. Many asylum seekers are without shelter (estimated around 2,050 persons in October 2022 (personal communication L Bruggeman, February 2023), most of them living on the street or some in squats or private housing.

## Outbreak detection

At the end of August 2022, Austria posted a public health alert in the European surveillance portal for infectious diseases (EpiPulse) [20], describing three clusters of diphtheria cases among asylum seekers. Discovery of cases in other European countries quickly followed. In response to this, Belgian regional health authorities and services responsible for asylum seekers were contacted and the first case, diagnosed in March 2022, was retrospectively linked to this European outbreak. The index case in Belgium was an Afghan asylum seeker with cutaneous diphtheria. More cases were diagnosed among asylum seekers in Belgium in the following weeks.

The aim of this study was to describe the epidemiological and microbiological aspects of the outbreak of *C. diphtheriae* infection among asylum seekers in Belgium, as well as the outbreak management by the authorities and humanitarian aid associations. The adaptations of the Belgian or European guidelines on case management and control are also described.

## Methods

### Case definitions

Cases included in this outbreak were asylum seekers in Belgium with laboratory confirmation of toxin-producing *C. diphtheriae* between 1 March and 31 December 2022. In addition, suspected cases of cutaneous diphtheria were defined as any asylum seeker with a skin lesion.

The definition of close contacts was roughly similar in all regions of Belgium and mainly included people living in the same household or having been in direct contact with wound or oropharyngeal secretions [21,22].

### Setting and data source

In Belgium, two structures, the Risk Assessment Group (RAG) and the Risk Management Group (RMG) were created in 2007 to comply with the requirements of the International Health Regulations of the World Health Organization (WHO) [23] and the European Union decision number 1082/2013/EU on the serious cross-border threats to health [24].

The RAG is chaired by the Belgian Institute of Public Health and includes permanent members from the federal and regional health administrations, as well as external experts on a specific topic. At the end of September 2022, the RAG assessed the risk of diphtheria for the Belgian population and asylum seekers and gave recommendations to the RMG. In the RMG, regional and federal health authorities of administrative and political level are represented. The task of the RMG is to convert the recommendations into decisions and actions to contain an outbreak. As regional authorities are responsible for the prevention of infectious diseases, each regional authority was responsible for the outbreak management in their respective region. The RMG coordinated the work.

We used a data collection form developed by the European Centre for Disease Prevention and Control (ECDC) for this outbreak, including demographic, epidemiological and microbiological variables. Upon mandatory notification by clinicians or laboratories, the regional health authorities collected demographic and epidemiological information. Information was obtained through contact with healthcare providers of the cases as well as through interviews (in-person or by phone) of cases. If available, interpreters were used in interviews. Regional health authorities sent information to the National Public Health Institute (Sciensano, Brussels) through Microsoft Excel files. Microbiological information was provided by the National Reference Centre (NRC), Universitair Ziekenhuis Brussel, Vrije Universiteit Brussel, Brussels. All Belgian laboratories are asked to send isolates of *Corynebacterium* species to the NRC for confirmation of toxin production and additional testing. Information on age, sex, vaccination status, place of residence and test results was transferred by the NRC to the public health institute via standardised secured web transfer. Data from NRC and health authorities were then compiled to a combined dataset. In case of inconsistencies or missing data, the data providers were contacted to verify (un)availability of the data. Information on measures taken within the reception facilities was obtained from Fedasil and from the regional health authorities. All the epidemiological and microbiological results were shared with ECDC to conduct an analysis of the outbreak at European level.

Data were stored and analysed in SAS software (SAS Institute, Cary, the United States) and Excel.

### Microbiological analyses

At the NRC, samples were cultured on blood agar and selective tellurite agar, incubated aerobically at 35°C for a maximum of three days. Isolates sent from other laboratories and suspected colonies from the samples cultured at the NRC were species identified using Matrix Assisted Laser Desorption lonization (MALDI-TOF) with the MALDI Biotyper system (Bruker, Germany).

A conventional PCR targeting the toxin gene and the RNA polymerase subunit β-gene (rpoB) was performed on all confirmed *C. diphtheriae* isolates. An in-house PCR based on Hauser et al. was used, with slight primer modifications [25]. Production of diphtheria toxin was confirmed using the modified Elek test [26].

Antimicrobial susceptibility to penicillin, erythromycin, clindamycin and rifampicin was determined using Etest (bioMérieux, France). The most recent European Committee on Antimicrobial Susceptibility Testing (EUCAST) guidelines were followed for interpretation [27].

Whole genome sequencing (WGS), including a core genome MLST analysis, was performed. The first nine isolates were sequenced at the Brussels Interuniversity Genomics High Throughput core (www.brightcore.be), using Illumina technology and the Nextera XT DNA Library Prep Kit (Illumina, the United States). The other 16 isolates were sequenced by IZSAM G. Caporale (Teramo, Italy), using Illumina technology and the Illumina DNA Prep Kit. The phylogenetic tree was created using the Bigsdb GrapeTree plugin [28,29].

## Results

### Outbreak outline

In total, 25 cases of toxin-gene bearing *C. diphtheriae* infection 23 cutaneous, one respiratory and one cutaneous and respiratory, were confirmed by the NRC between March and December 2022.

**Figure 1:**
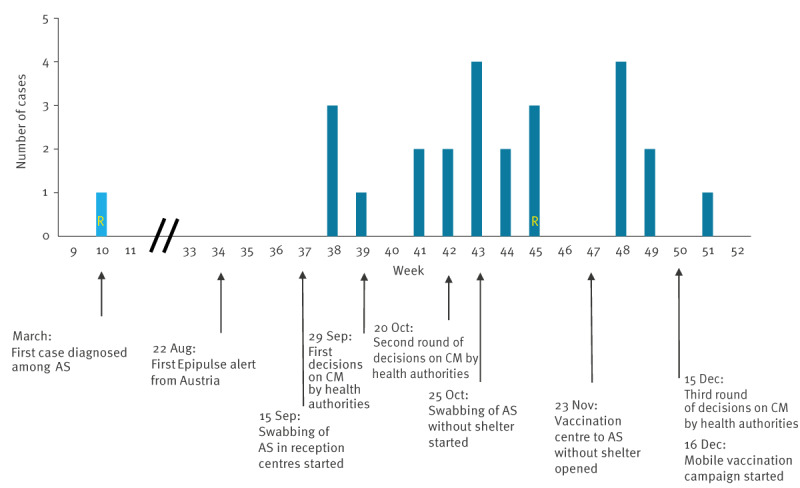
Timeline of diphtheria case notifications and control measures taken in an outbreak among asylum seekers, Belgium, March–December 2022 (n = 25)

AS: asylum seeker; CM: control measures; R: antimicrobial resistant isolate.

The epidemic curve of this outbreak, including the major interventions to manage it, is presented in Figure 1.

EpiPulse is an online portal, managed by the European Centre for Disease Prevention and Control (ECDC), for European public health authorities and partner organisations to collect, analyse, share, and discuss infectious disease data for threat detection, monitoring, risk assessment and outbreak response.

Decisions on control measures were taken by the Risk Management Group. More details about these decisions are available in Table 3.

Demographic, clinical and microbiological information about the cases is presented in Table 1.

**Table 1:** Demographic and clinical characteristics of diphtheria cases in an outbreak among asylum seekers, Belgium, March–December 2022 (n = 25)

Characteristics	n
Sex	
Male	24
Female	1
Age group	
< 18 years	6
18–25 years	16
26–35 years	2
36–45 years	1
Resident in a refugee centre	
Yes	11
No	11
Unknown	3
Place of residence (region)^a^	
Brussels	21
Flanders	4
Clinical manifestation	
Cutaneous	23
Respiratory	1
Cutaneous and respiratory	1
Outcome	
Alive	25
Death	0
Vaccination status	
Unknown	17
One dose^b^	7
Two doses^b^	1
Country of origin	
Afghanistan	21
Syria	3
Unknown	1

^a^ Place of the first notification of the case to a regional authority.

^b^ Vaccination on arrival in Belgium.

Most cases were young males from Afghanistan with a clinical picture of cutaneous diphtheria and residing in Brussels (Table 1). The vaccination status was frequently unknown due to missing vaccination records. Data on migration routes were collected for only one case (not presented). No deaths were reported. No secondary cases were detected among the identified close contacts or within a reception centre. All cases had recently arrived in Belgium, were either asylum seekers or going to apply for asylum.

Two cases were diagnosed and hospitalised for respiratory diphtheria. The first one, an unaccompanied minor, had arrived from a neighbouring country the day before their condition deteriorated. They presented tonsillar pseudomembranes and croup and developed myocarditis, requiring the administration of DAT. The second case of respiratory diphtheria was also an unaccompanied minor living in a reception centre for minors and presented with a mild disease. No DAT was administrated, and they fully recovered.

### Microbiological analyses

**Figure 2:**
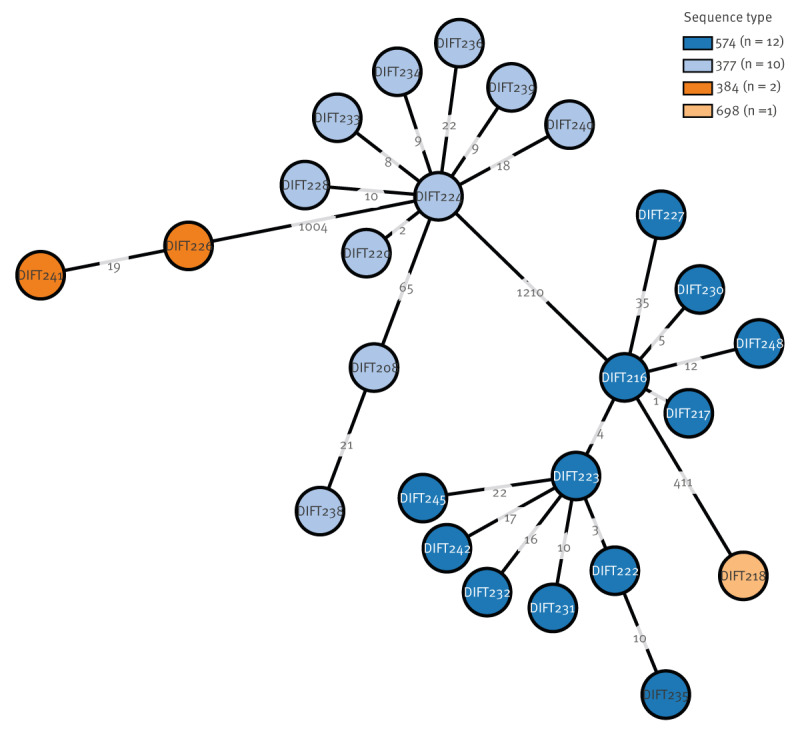
Phylogenetic tree of *Corynebacterium diphtheriae* isolates from asylum seekers, Belgium, March–December 2022 (n = 25)

DIFT: *Corynebacterium diphtheriae*; ST: sequence type.

Most (n = 22) of the outbreak isolates were of sequence type (ST) 574 or 377, two belonged to ST698 and one to ST384 (Figure 2).

The numbers in the bubbles refer to the isolate identification number. The branch labels represent the number of allelic mismatches between strains using the Bigsdb cgMLST scheme.

The phylogenetic tree was created using the Bigsdb GrapeTree plugin. Branch lengths are presented in logarithmic scale.

In early January 2023, after other European countries warned about erythromycin resistance [16], minimal inhibitory concentration values (MIC) were reinterpreted using the new EUCAST breakpoints published in 2023 [27]. Applying these breakpoints, two isolates were determined erythromycin resistant: one from March 2022 and another from November 2022. While both isolates belonged to ST377, they diverged from the other ST377 isolates by significantly more than 25 allelic mismatches, the threshold for a genomic cluster [30]. Macrolide resistance was confirmed by the presence of the *erm* gene in both.

### Outbreak control measures

All decisions taken by the Belgian authorities related to this outbreak are described in Table 2.

**Table 2:** Issues raised and decisions taken by the Risk Management Group^a^ in an outbreak of diphtheria among asylum seekers, Belgium, March–December 2022

Issue and date	Decision
29 September (week 39)	
Risk of diphtheria among unvaccinated or incompletely vaccinated staff in contact with asylum seekers	Checking vaccination status and vaccination of staff in contact with asylum seekers
Risk of diphtheria among unvaccinated or incompletely vaccinated asylum seekers	Free vaccination for all asylum seekers
No more DAT available in Belgium and legal problems to supply and store it	Find solutions to order and store DAT (order with a neighbouring country/constitution of a strategic stock)
Diphtheria is a rare disease and therefore decreased awareness among healthcare workers	Information on the epidemiological situation and existing guidelines to doctors
20 October (week 42)	
Missing diagnosis and treatment of asylum seekers without shelter	Improving the capacity of diagnosis through wound swabs and reimbursement of laboratory tests from suspected cases of cutaneous diphtheria seeking medical care, irrespective of the registration status
	Empirical antimicrobial treatment of suspected cases
Lack of vaccination of asylum seekers without shelter	Additional vaccination of asylum seekers without shelter
Delays to complete vaccination schedules of adults and children in reception centres	Need to find solutions to speed up the completion of the vaccination schedules
15 December (week 50)	
Unsustainable delivery of DAT provided on two occasions by the Netherlands	Speed up outstanding orders and evaluate need for additional emergency order
DAT order in progress in Belgium	
Suspected diphtheria among unvaccinated staff in contact with asylum seekers	Reminder about urgent vaccination of staff in contact with asylum seekers

DAT: diphtheria antitoxin.

^a^ The minutes from the meetings of the Risk Management Group can be found in the reference list [40-42].

#### Case finding and diagnosis

Following the public health alert from Austria and ECDC about the diphtheria outbreak among asylum seekers, Fedasil was quickly informed of the risk of diphtheria cases. From mid-September, testing for *Corynebacterium* from skin lesions was systematically introduced in the reception centres, with particular attention for asylum seekers from Afghanistan and Syria. In the absence of respiratory symptoms, a pharyngeal swab was only taken when the pathogen had been detected from skin lesion. Any asylum seekers showing clinical signs of respiratory diphtheria were promptly referred to the nearest hospital to confirm or exclude the diagnosis and receive appropriate treatment. The number of swabs taken in reception centres is unknown.

Due to an increased influx of asylum seekers, many asylum seekers were not accommodated in any official reception centre, which resulted in difficulties in case finding. As these persons needed care for various health issues, non-governmental humanitarian aid associations such as Médecins Sans Frontières (MSF) set up medical consultations in early October 2022. Due to difficulties in further medical follow-up, skin lesions were initially empirically treated with azithromycin for 3 days, without microbiological testing or registration of suspected cases. After a second assessment on 20 October 2022, Fedasil agreed on funding microbiological testing of all asylum seekers who presented with wounds at medical consultations. From then, swabbing of suspected skin lesions was initiated, primarily for epidemiological purposes rather than for individual case management. In total, MSF empirically treated 147 suspected cases of diphtheria. Of these, 111 were sampled, which resulted in eight (7.2%) confirmed cases. Some asylum seekers had scabies skin lesions with bacterial superinfection leading to atypical clinical presentations which was challenging for diagnosis.

At the end of November, the Brussels Infection Prevention and Control Unit planned a screening of a large building (squat) where over 600 people from various origins were living unofficially. Unfortunately, only ca 200 individuals, mainly of Afghan origin, were present during the daytime visit. A wound swab was taken from 12 of 14 asylum seekers with skin lesions (two refused to be tested). Toxigenic *C. diphtheriae* was detected in one of these samples.

#### Treatment and isolation of cases

As the stocks of diphtheria antitoxin available in Belgium had expired, the RAG recommended on 29 September 2022 that DAT should be made available, and an order was placed on 15 December 2022 (Table 2).

For confirmed cases residing in reception centres, treatment with clarithromycin 2 × 500 mg/d for 14 days was initiated, as recommended in national guidelines [31].

Suspected cases of cutaneous diphtheria in asylum seekers residing outside reception centres and who consulted the humanitarian aid posts, were treated with azithromycin (500 mg for 3 days) without waiting for laboratory results. The shorter treatment duration of 3 days was chosen to increase compliance.

Isolation facilities for cutaneous cases were limited, both inside reception centres and for those without shelter. While ECDC recommends isolation of all confirmed cases of respiratory or cutaneous diphtheria until the elimination of the organism is demonstrated by two negative cultures obtained at least 24 hours apart after completion of antimicrobial treatment [3], in our setting, only covering the wound was possible, and no swabs were taken to confirm the elimination of the pathogen.

The difference in case management between asylum seekers inside reception centres and those without shelter is summarised in Table 3.

**Table 3:** Investigation steps and interventions inside and outside a reception centre in an outbreak among asylum seekers, Belgium, March–December 2022

Interventions	Activities inside a reception centre	Activities outside a reception centre (asylum seekers without shelter)
Case finding and diagnosis	Swab of suspected cases: wound swab and pharyngeal swabs if a positive wound swab	Before October: no case finding and no diagnosis available
		From early October: empirical treatment of suspected cases and no swabbing^a^
		From 25 October: swabbing and empirical treatment of suspected cases^a^
Treatment	Clarithromycin 2 x 500 mg/day for 14 days if *C. diphtheria*e detected	Azithromycin 1 x 500 mg/day for 3 days, without waiting for detection results
Isolation	Coverage of skin lesion	Coverage of skin lesions
	If pharyngeal carriage: respiratory droplet isolation until 24 h after start of antimicrobial treatment	
Contact tracing and antimicrobial prophylaxis	In case of respiratory diphtheria or pharyngeal carriage of an index case → 3 days of azithromycin 500 mg/day for all close contacts	Not possible
Vaccination^b^	0–≤ 6 years: full vaccination through early childhood consultations	Through vaccination centre or mobile vaccination campaign (no selection according to age or country of origin)
	> 6–< 12 years: full vaccination through school healthcare	
	≥ 12 years: first dose on the day of registration and other doses at the reception centre	

^a^ Available only for people going to the medical consultation.

^b^ Catch-up vaccination was once organised for a respiratory case inside a reception centre for unaccompanied minors.

#### Prophylaxis in close contacts

Antimicrobial prophylaxis in close contacts of cases with cutaneous diphtheria was only initiated in case of pharyngeal carriage of the case. All minors (n = 75) living close to each other and the staff in a reception centre for non-accompanied minors, where a case with respiratory diphtheria resided, received azithromycin 500 mg/day for 3 days as a prophylaxis. A catch-up vaccination of these contacts, including the staff, was organised, but targeted catch-up vaccination of other contacts was not possible because of understaffing of medical staff at the reception centre.

The other case with respiratory diphtheria had only just arrived from a neighbouring country when the symptoms began, and the countries’ authorities were contacted to organise contact tracing if possible.

More details about management of cases and their contacts inside reception centres are available in Table 3.

For cases living on the streets, no contact tracing was possible.

#### Vaccination campaigns

To vaccinate asylum seekers without shelter, a vaccination centre in Brussels was opened mid-November 2022 and diphtheria-tetanus-pertussis (dTap) and polio vaccinations were offered. Additionally, a one-week mobile vaccination campaign was organised by MSF during December 2022. The campaign intended to reach out to those who would not have come to the vaccination centre, i.e. persons living in unofficial accommodations or those who were difficult to mobilise. By the end of December, 362 asylum seekers had received the first dose of dTpa and polio inside the vaccination centre and an additional 443 asylum seekers during the mobile vaccination campaign. Additional doses were offered through a Refugee Medical Point organised by the Red Cross that opened in mid-January 2023.

Additionally, all staff inside reception centres were informed about diphtheria and encouraged to check their vaccination status.

## Discussion

We describe the management of an outbreak of diphtheria among asylum seekers in Belgium. The outbreak affected also many other European countries [3,16,17] and occurred mainly among young males from Afghanistan and Syria presenting with cutaneous diphtheria. Transit through a country or countries along the Balkan route has been previously identified as the most likely source of infection for these migrants [16,17].

In 2015, ECDC published an expert opinion on the public health needs of irregular migrants, refugees or asylum seekers across the EU's southern and south-eastern borders [32]. The document highlights the need of reception centres for newly arrived migrants, to avoid crowding and ensure good sanitation and hygienic conditions. There are also options for screening for communicable diseases and vaccination. Admittedly, the increasing number of people entering the EU, and particularly Belgium, as asylum seekers and irregular migrants, has challenged authorities. Our outbreak management was hindered by the numerous asylum seekers without shelter, the difficulties to order DAT and the understaffing inside reception centres. In addition, as at least some cases likely got infected outside Belgium or EU, local interventions had a limited effect on the incidence of new cases.

During this outbreak, national recommendations for curative care and prophylactic treatment had to be adapted. In a population with poor living conditions, compliance is often reduced, and regimens should be as short as possible with a minimum of daily doses. During the diphtheria outbreak among Rohingya refugees in Bangladesh, a once-a-day administration of azithromycin was therefore preferred over multiple daily doses of erythromycin or penicillin, seemingly without negative consequences on outcome [33]. In the Bangladesh outbreak, adherence to chemoprophylaxis was only 55% on day 3, and the prophylactic regimen was shortened to a 3-day course. Shortened treatment regimens carry a risk of treatment failure, potentially driving antimicrobial resistance. However, as shown by the Bangladesh data, it is likely that compliance with longer treatment regimens would have been low for mild cutaneous cases, thus removing the theoretical advantage of longer treatment regimens. Data to support the preferred duration of treatment with azithromycin are lacking [34]. However, as azithromycin has an elimination half-life of 2–4 days and concentrations in infected tissues exceed serum concentrations, 500 mg/day for 3 days is the Belgian recommended dosage for uncomplicated skin infections [35]. This regimen was thus a logical choice for empirical treatment of skin infections. At the onset of the outbreak, known macrolide resistance for *C. diphtheriae* was rare, as shown by analysis of a large dataset, mostly from France, with only 2.5% of all clinical isolates resistant to erythromycin [36]. We also deviated from national and ECDC recommendations, especially for asylum seekers without shelter, regarding isolation and contact tracing [3]. As implementing these guidelines poses practical and logistical challenges to implement in these specific circumstances, a pragmatic approach was chosen instead.

Our outbreak control relied heavily on vaccination. The campaigns for asylum seekers without shelter were rather successful, as indicated by the number of vaccinations given. However, mass vaccination alone might not suffice to stop the outbreak [5,16]. Vaccination has been estimated to sufficiently interrupt transmission in 27% of outbreak settings [5]. This improves to 70% with rapid antimicrobial treatment of 90% of symptomatic cases because antimicrobial treatment accelerates the clearance of colonisation and symptomatic cases are more contagious than asymptomatic cases. Thus, mass antimicrobial prophylaxis, contact tracing and case isolation have been considered critical to interrupt transmission [5]. Indeed, the UK Health Security Agency recommends mass vaccination and mass antimicrobial prophylaxis in high volume reception settings where individual case and contact management is not possible [37]. However, in our setting, mass antimicrobial prophylaxis was neither feasible nor sure to be effective, as it did not concern a closed community. Moreover, increased use of macrolides risks driving resistance [38].

Despite the overall limited options for outbreak control, we did not find clear epidemiological evidence for local transmission. No secondary cases were detected inside reception centres and few confirmed cases were reported. We acknowledge, however, the limitations in data quality, due to a combination of factors. The large number of actors involved in managing this outbreak, chronic understaffing inside reception centres and high numbers of asylum seekers without shelter all contributed to suboptimal data collection. The number of diagnosed cases will thus be underestimated. First, a wound swab was only taken from asylum seekers without shelter if they actively seeked healthcare or were present during the screening in the squat. Second, tracing and screening of asymptomatic contacts were very limited. Indeed, no asymptomatic cases were reported in this Belgian outbreak, whereas Switzerland [17] and Germany [16] observed asymptomatic carriers among close contacts. Belgium is, however, not the only country to not have reported asymptomatic cases: the variation in numbers of reported cases from other EU countries [3] is likely to reflect different testing practices. Finally, it is sometimes difficult to isolate *C. diphtheriae* from wound cultures, as the flora is often polymicrobial.

Nevertheless, there are also some elements to support our observation that the outbreak was largely controlled and that new cases were the result of repeated importation rather than of local circulation. Firstly, no new cases were detected between end of December and May 2023. Secondly, reported cases belonged to four different STs, which suggests independent introductions and transmission chains. The Belgian isolates belonged to STs identified in other European countries, as shown by results from an ECDC-led pan-European consortium which analysed 366 strains from 10 countries, including Belgium [39]. Some ST377 and 574 isolates found in Belgium were closely related, but we could not find an epidemiological link between these cases. Indeed, there was no temporal or geographical link: the cases had sometimes recently arrived in Belgium or were diagnosed with several weeks delay between them or the cases were in reception centres in different regions. The close similarity (short branch length) could indicate either undetected transmission in Belgium (e.g. as people waited near the registration centre to apply for asylum) or transmission along a common migration route or a reception centre abroad.

Based on our experience during this outbreak, we learnt some lessons. Clearly, the lack of shelters for arriving asylum seekers increased health risks and complicated outbreak management. In our opinion, urgent solutions should be sought, both at national and EU level. Fragmentation of responsibilities between many different actors made management complex. Having a clearly identified central coordinating body would be important in such an outbreak situation. Preparedness was not optimal, with e.g. no availability of DAT in Belgium at the start of the outbreak. Also, although, all incoming asylum seekers should be offered vaccination upon arrival, the offer was limited or delayed. For instance, vaccination schedules were not completed, or migrant children were vaccinated through school health services after a delay of several months. At the time of writing, solutions for carrying out the vaccination within the recommended timeframe and schedule, both for adults and children, were still under discussion. Moreover, it is important to improve data on vaccination coverage. It was difficult to have an overview on the vaccination status of asylum seekers and staff due to a lack of harmonised vaccination records. Given the high mobility of asylum seekers between various reception centres in Belgium and beyond, administered doses should ideally be recorded in a uniform European vaccination card. While it seems neither feasible nor desirable to have guidelines tailored to each specific situation or population, we believe it is important to consider logistical and budgetary implications of certain interventions and allow pragmatic adaptations or prioritisation of some measures in complex outbreaks like this one. Finally, as diphtheria has become a rare disease, many clinicians lack experience with the disease. Therefore, it is important to raise awareness on diphtheria so that the disease can be adequately diagnosed and rapidly treated.

Unfortunately, recent events in Belgium have shown that these lessons learnt have not yet been fully implemented. At the end of June 2023, three respiratory diphtheria cases were reported. All cases occurred in related minors of a family living in a reception centre. Diagnosis of the index case was delayed, and the patient did not receive DAT and sadly died from toxic complications. Although the family arrived in Belgium in early 2020, only the youngest child had received full primary vaccination against diphtheria. As there was no recent travel history, the source of the infection appears to have been undetected transmission in Belgium.

## Conclusion

This diphtheria outbreak comprising 25 individuals was difficult to manage, pragmatic adaptations were made and there remained a risk for undetected further spread. We identified several areas for improvement and in Belgium, more resources should be allocated to asylum seekers. A rapid vaccination of asylum seekers upon arrival in Belgium is necessary. Solutions also need to be found at the international level for the management of this outbreak involving asylum seekers in several European countries, such as detecting and supporting countries where asylum seekers are becoming infected, such as the Balkan route, to contain the spread of diphtheria. Improving vaccination coverage in the countries of origin is also relevant in this context.
